# Use of artificial intelligence to track platelet-rich plasma treatment outcomes in females with nonscarring alopecia: A case series

**DOI:** 10.1016/j.jdcr.2024.02.037

**Published:** 2024-03-27

**Authors:** Ora Raymond, Javed Shaik, Katelyn Rypka, Ronda S. Farah, Gretchen Bellefeuille, Maria Hordinsky

**Affiliations:** a Department of Dermatology, University of Minnesota Medical School, Minneapolis, Minnesota; b Department of Dermatology, Minneapolis Veterans Affairs Medical Center, Minneapolis, Minnesota

**Keywords:** alopecia, androgenetic alopecia, artificial intelligence, platelet-rich plasma, trichoscopy

## Introduction

Platelet-rich plasma (PRP), a minimally invasive procedure, has shown promising results in the treatment of androgenetic alopecia (AGA).^1^^,^^2^ During this procedure, patients’ platelets are injected into the scalp.^1^^,^^2^ Over the course of 3 PRP treatments (typically given once per month for 3 consecutive months) improved hair growth has been reported.^1^^,^^3^^,^^4^ However, objectively assessing PRP efficacy is challenging. Current methods to assess alopecia treatment efficacy include global photographic comparison, manual trichoscopy, physician assessment, and patient self-assessment.^2^, ^3^, ^4^ These approaches are considered to be qualitative and subjective. To provide automatic quantitative and qualitative data to assess hair density and scalp health during clinical visits, we now routinely utilize photography with artificial intelligence (AI) technology. Herein, we report on the use of AI-derived data to evaluate the efficacy of PRP treatments in 3 female patients with AGA.

## Methods

Three White female patients with AGA (Ludwig stage I-II) diagnosed and treated by a board-certified dermatologist at the University of Minnesota M Health Fairview Dermatology Clinic were included in this case series. PRP treatments occurred between October 30, 2020 and February 11, 2022. Patients A, B, and C were ages 68, 30, and 72 at baseline, respectively. Pharmacological AGA treatment regimens were not standardized nor discontinued prior to PRP baseline treatment. However, AGA treatments were at maintenance dose to reduce confounding factors and hair shedding associated with the initiation of medications like topical minoxidil. Two minor exceptions to maintenance include patient A decreasing spironolactone dosage from 100 mg twice daily to 50 mg twice daily at their fourth PRP due to scalp hair improvement and Patient C switched from ketoconazole to ciclopirox shampoo (Table I). No patients had undergone hair transplantation nor were any pregnant or breastfeeding during the treatment series.

**Table I:** Patients’ A to C prescribed hair loss treatments prior and following first platelet-rich plasma procedure

Patient	Hair loss treatments at initiation and during PRP therapy	Frequency	Start date	First PRP date
Patient A	Photobiomodulation	Three times per wk	9/25/2018	10/30/2020
	Spironolactone	100 mg twice daily	8/19/2019	
		Decreased to 50 mg twice daily	7/9/2021	
Patient B	Photobiomodulation	Three times per wk	1/24/2017	1/8/2021
	Spironolactone	50 mg daily	10/12/2019	
	Clobetasol shampoo	Once per wk	1/08/2021	
	Ketoconazole 2% shampoo	Two-3 times per wk	1/08/2021	
	Fluocinolone oil	As needed	9/17/2021	
Patient C	Fluocinolone oil	As needed	12/17/2013	2/26/2021
	Topical minoxidil 5% foam	Daily	2/25/2013	
	Ketoconazole 2% shampoo	Twice weekly	3/26/2013	
		Discontinued	10/21/2021	
	Ciclopirox shampoo	Twice weekly	10/21/2021	
	Photobiomodulation	Three times per wk	3/26/2013	
	Finasteride	1 mg daily	8/08/2016	

*PRP*, Platelet-rich plasma.

The HairMetrix D200 EVO (Parsippany) dermatoscope captures images that are 15x magnified and utilizes AI to yield quantitative results such as hair fiber diameter (μm), hairs per follicular unit, and follicle counts by cm^2^ by automatically selecting hair fibers.^5^ Images of the frontal anterior, midscalp, and vertex scalp were captured at baseline and prior to sequential PRP injections. Imaging sites were standardized between each patient’s frontal anterior, midscalp, and vertex scalp. The distance between the glabella and scalp photography site to the nearest quarter centimeter was measured and an AI software-generated diagram was used to monitor the location of the images.

PRP was prepared using the Eclipse PRP System (Eclipse aesthetics). Twenty-two milliliters of a patient’s blood was drawn. Tubes were centrifuged at 1500× g for 10 minutes and PRP was prepared following the manufacturer's protocol. PRP injection sites varied slightly according to patient preferences, although in all cases the entire frontal anterior, midscalp, and vertex scalp was treated.

PRP treatment frequency varied depending on patient, physician, and clinic availability. The average interval between PRP treatments was 13 weeks for patient A, 10 weeks for patient B, and 8 weeks for patient C. All images underwent manual hair fiber diameter selection, completed by 1 researcher, following automatic AI detection to correct false positives and negatives.

## Results

**Fig 1:**
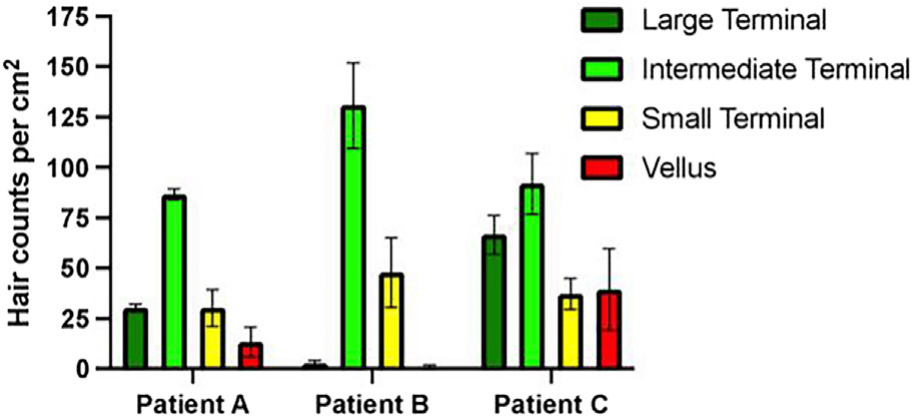
Androgenetic alopecia baseline densities of different hair fibers measured by AI trichoscopy in patients A to C. The average densities from frontal anterior, mid, and vertex scalp of large terminal (>90-μm hair base width), intermediate (60-90 μm) and small (30-60 μm) along with vellus hair fibers (<30 μm) as reported by the AI device for patients A-C is shown. Baseline densities of large terminal and vellus hair fibers are unique to each of the patients. *Error bars* indicate standard error of the mean.

The device provided baseline densities of the terminal [large (>90-μm hair base width), intermediate (60-90 μm), and small (30-60 μm)] and vellus hair fibers (<30 μm) for each patient. Patient C had the highest number of large terminal and vellus hair fibers, averaging 67 fibers/cm^2^ and 40 fibers/cm^2^, respectively, across frontal anterior, midscalp, and vertex scalp sites. Patient A had an intermediate count of large terminal (30 fibers/cm^2^) and vellus hair (13 fibers/cm^2^) while patient B had the least number of these hair fibers (<3 fibers/cm^2^) (Fig 1).

**Fig 2:**
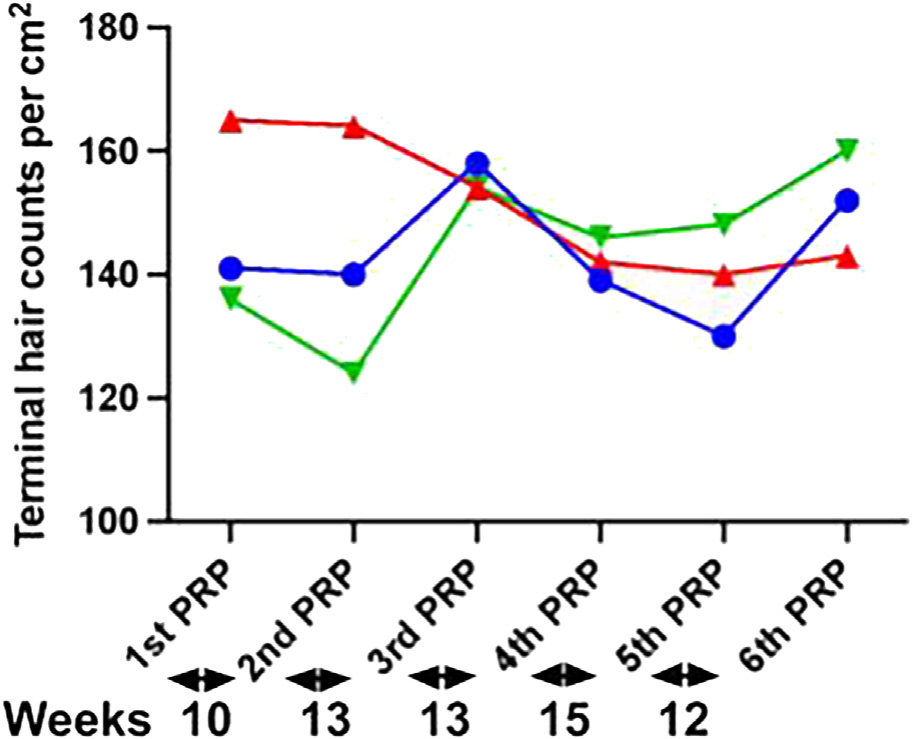
Androgenetic alopecia real-time changes in terminal hair counts during PRP treatments measured by AI trichoscopy in patient A. Real-time quantitative total terminal hair fiber (large, intermediate, and small terminal fibers) per cm^2^ counts from the frontal anterior (*blue*), mid scalp (*red*), and vertex (*green*) regions at baseline/first PRP and at PRP treatments shown under the graph for patient A. Baseline measurements were taken at the time of the first PRP treatment. The time interval in weeks between the treatments is indicated under the graph. *PRP*, Platelet-rich plasma.

**Fig 3:**
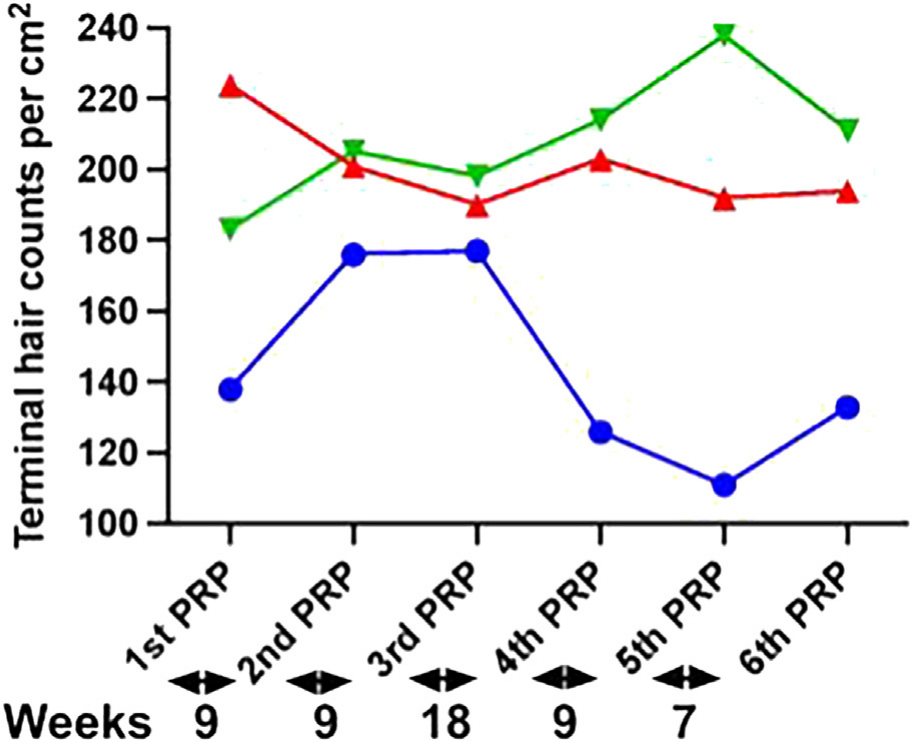
Androgenetic alopecia real-time changes in terminal hair counts during PRP treatments measured by AI trichoscopy in patient B. Real-time quantitative total terminal hair fiber (large, intermediate, and small terminal fibers) per cm^2^ counts from the frontal anterior (*blue*), mid scalp (*red*), and vertex (*green*) regions at baseline/first PRP and at PRP treatments shown under the graph for patient B. Baseline measurements were taken at the time of the first PRP treatment. The time interval in weeks between the treatments is indicated under the graph. *PRP*, Platelet-rich plasma.

**Fig 4:**
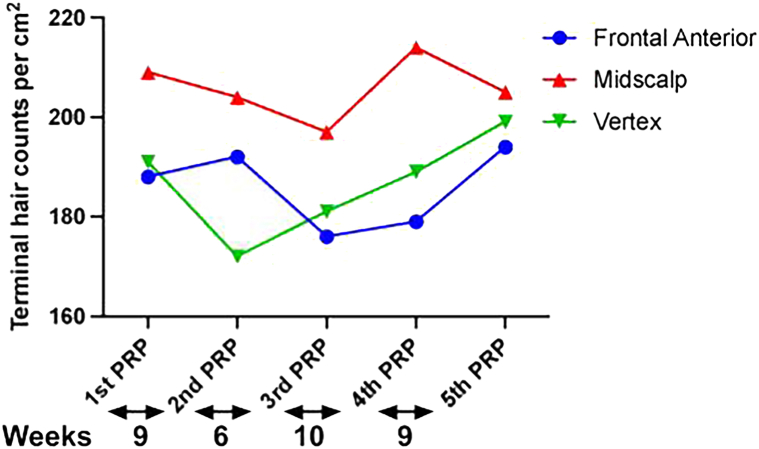
Androgenetic alopecia real-time changes in terminal hair counts during PRP treatments measured by AI trichoscopy in patient C. Real-time quantitative total terminal hair fiber (large, intermediate, and small terminal fibers) per cm^2^ counts from the frontal anterior (*blue*), mid scalp (*red*), and vertex (*green*) regions at baseline/first PRP and at PRP treatments shown under the graph for patient C. Baseline measurements were taken at the time of the first PRP treatment. The time interval in weeks between the treatments is indicated under the graph. *PRP*, Platelet-rich plasma.

The terminal hair (>30 μm) count improved by 7% and 18% in the frontal anterior and vertex scalp, respectively, for patient A at the end of 5 PRP treatments (Fig 2). A dramatic increase in large terminal hair fibers was measured for patient A with an increase from 27 to 54 fibers in the frontal anterior scalp and from 30 to 51 in the vertex scalp (data not shown). The device reported an improvement of 28% in terminal hair fiber count of the frontal anterior scalp after the first and second PRP treatment for patient B. However, a decline was noticed after the third PRP session that was followed by an upward trend at the end of fourth and fifth PRP (Fig 3). The AI device indicated an improvement of 15% in terminal hair fiber count of the vertex scalp for patient B at the end of 5 PRP treatments (Fig 3). The terminal fiber count for patient C increased in the vertex scalp following an initial decline but remained stable across the frontal anterior and midscalp regions (Fig 4). There was an increase in large terminal hair fibers at the end of 4 PRP treatments for patient C with a 30% and 15% increase in the midscalp and vertex regions, respectively (data not shown).

## Discussion

AI has been demonstrated to provide quantitative data to support evidence-based clinical decision making in dermatology.^6^, ^7^, ^8^ The results of this study present a potential solution to current difficulties in evaluating PRP efficacy by providing quantitative data for hair fiber number and diameter. While improvements in hair density during the treatment series appeared slight to moderate, the AI device measurements indicated an increase in large terminal hair fibers. The location-specific quantitative analysis of hair fibers and hair density using the AI software is noninvasive and less laborious compared to methods like hair clipping or tattooing. Additionally, each magnified image offers important qualitative information such as presence of erythema, scale, and telangiectasias that cannot always be appreciated to the same degree clinically.

The real-time AI hair measurements can help inform physician and patient decisions regarding optimal scalp PRP injection intervals and sites between subsequent PRP treatments. Patient B’s decline in terminal hair fiber count of the frontal anterior scalp following second PRP treatment may be attributed to the 18-week gap between the second and third PRP treatment. When shorter treatment intervals resumed, improvement in terminal hair fiber density was observed. The AI device also identified scalp sites that showed no improvement or even a decline in terminal hair fiber counts. These included the mid scalp region for patients A and B and the frontal anterior region for patient C. Therefore, future PRP injections that target these sites were recommended with the goal of achieving consistent improvements across all scalp regions.

### Limitations

This study was single centered with 1 AI device. The sample size was small. The PRP system and protocol were consistent for all treatments; however, PRP session intervals were not standardized due to real-life clinic scheduling challenges. Additionally, selection of frontal anterior, midscalp and vertex scalp sites for AI imaging may vary minimally between treatments.

## Conclusions

AI technology provides quantitative data by which treatment progress can be objectively measured. In this retrospective series, hair fiber density reported by the AI device highlights early trends in determining focal PRP efficacy. We conclude that AI technology provides an improved and objective approach to the assessment of clinical response to treatments such as PRP. The use of this technology can be standardized in the clinical setting as well as in studies of treatments for hair loss.

## Conflicts of interest

None disclosed.
